# Mediterranean Diet-Based Interventions to Improve Anthropometric and Obesity Indicators in Children and Adolescents: A Systematic Review with Meta-Analysis of Randomized Controlled Trials

**DOI:** 10.1016/j.advnut.2023.04.011

**Published:** 2023-04-29

**Authors:** José Francisco López-Gil, Antonio García-Hermoso, Mercedes Sotos-Prieto, Iván Cavero-Redondo, Vicente Martínez-Vizcaíno, Stefanos N. Kales

**Affiliations:** 1 Navarrabiomed, Hospital Universitario de Navarra (HUN), Universidad Pública de Navarra (UPNA), IdiSNA, Pamplona, Navarra, Spain; 2 Department of Environmental Health, Harvard T.H. Chan School of Public Health, Boston, Massachusetts, United States; 3 One Health Research Group, Universidad de Las Américas, Quito, Ecuador; 4 Department of Preventive Medicine and Public Health, School of Medicine, Universidad Autónoma de Madrid, IdiPaz (Instituto de Investigación Sanitaria Hospital Universitario La Paz), Madrid, Spain; 5 CIBERESP (CIBER of Epidemiology and Public Health), Madrid, Spain; 6 IMDEA-Food Institute, CEI UAM + CSIC, Madrid, Spain; 7 Health and Social Research Center, Universidad de Castilla-La Mancha, Cuenca, Spain; 8 Facultad de Ciencias de la Salud, Universidad Autónoma de Chile, Talca, Chile

**Keywords:** obesity, overweight, lifestyle, eating healthy, youths, young population, preschoolers

## Abstract

To our knowledge, no systematic review with meta-analysis has separately synthesized the effects of Mediterranean diet-based interventions in children and adolescents in relation to the effects on anthropometric measures. A better understanding of the effects of Mediterranean diet-based interventions on anthropometric variables could facilitate their implementation in efforts to prevent obesity in the young population. The aim of the present meta-analysis was to evaluate the effects of Mediterranean diet-based interventions on anthropometric and obesity indicators among children and adolescents. Four databases were systematically searched (PubMed, Scopus, Web of Science, and Cochrane Database of Systematic Reviews), including all studies up until 15 March, 2023. Eligible articles were randomized controlled trials measuring the effect of an intervention based on the promotion of the Mediterranean diet and obesity-associated parameters. The effect size of each study was estimated by Cohen’s *d* for continuous variables or risk difference for categorical variables. Compared to the control group, the Mediterranean diet-based interventions showed small and significant reductions in body mass index (*d* = −0.14; 95% CI: −0.26, −0.01; *I*^2^ = 77.52%). Participants in the Mediterranean diet-based interventions had a significant reduction in the percentage of obesity (*risk difference* = 0.12; 95% CI: 0.01, 0.23; *I*^2^ = 84.56%) in comparison with the control group. Interventions had greater effects when aiming at participants with excess weight (that is, overweight or obesity), both for body mass index, waist circumference, waist-to-height ratio, percentage of obesity, and percentage of abdominal obesity. Mediterranean diet-based interventions have a significant effect on reducing the body mass index as well as reducing obesity in children and adolescents (aged 3–18 y). This trial was registered at PROSPERO as CRD42023386789.

**Statement of Significance**

- No systematic review with meta-analysis has synthesized the effects of Mediterranean diet-based interventions in children and adolescents in relation to the effects on anthropometric measures.
- Mediterranean diet-based interventions seem to be a useful tool in the interest of reversing the high prevalence of obesity.

## Introduction

Childhood obesity has been postulated as one of the most serious public health problems of the 21^st^ century due to its chronic adverse consequences [1]. Globally, excess weight (overweight or obesity) in childhood represents a major public health threat, especially in Europe, where the latest round of the Childhood Obesity Surveillance Initiative (COSI) [2], involving 33 European countries, indicated that 29% of children aged 7 to 9 y had excess weight according to WHO criteria (31% in boys and 28% in girls). Furthermore, the risk of morbidity and mortality in adult life increases in those with excess weight in childhood or adolescence [3]. For this reason, the member states of the WHO have committed themselves to prevent further increases in childhood obesity rates after 2025 [4].

The etiology of obesity is complex [5] and involves the interaction of social [6], biological [6], genetic [7], and environmental factors [6,8], among others [6]. Concerning genetics, between 40% and 70% of the variation in BMI could be attributable to hereditary factors [9]. Polygenic obesity is the most common, whereas single-gene (monogenic) obesity-related syndromes and defects account for <1% of childhood obesity [10,11]. Regarding environmental factors, the WHO has especially attributed 2 factors to the current increase in the prevalence of obesity: first, the consumption of high-calorie foods that are rich in sugar and fat has increased significantly; second, people are becoming more sedentary due to changes in work patterns, transportation methods, and the growing urbanization trend [12]. Furthermore, diet quality has been suggested to play a moderating role in the relationship between some health outcomes (for example, inflammatory biomarkers [13] and psychosocial health [14]) and excess weight. In addition, the recommended levels of physical activity, sedentary behavior, and sleep duration in adolescence have been related to a lower risk of abdominal obesity later in life [15]. Therefore, lifestyle modifications, including dietary modifications aimed at reducing total energy intake, increasing physical activity, and decreasing sedentary time, seem to be crucial for the control of body weight [3].

Among healthy dietary patterns, the Mediterranean diet (MedDiet) has been recognized worldwide due to its distinctive health benefits [16,17]. The MedDiet is characterized by a pattern rich in fruits and vegetables (seasonal), legumes, whole grains, nuts, and olive oil as the main dietary fat, with greater consumption of white or lean meats than of red or processed meats, moderate consumption of dairy products (cheese and milk), moderate consumption of fish and eggs, and intake of small amounts of wine with meals [18]. Supporting this notion, scientific evidence supports an inverse relationship between MedDiet and noncommunicable diseases (for example, cancer, metabolic syndrome, hypertension, or cardiovascular diseases) [19], as well as mortality [20], with some of the dietary components mentioned above having a substantial influence on this relationship [20]. Specifically, in young people, greater adherence to an MedDiet [16] has been associated with greater anti-inflammatory potential [21], physical fitness [22], or health-related quality of life [23]. However, despite being an evidence-based healthy pattern, a systematic review has pointed out the clear trend of decreasing MedDiet adherence in Mediterranean countries between 2004 and 2014, especially among children [24].

Despite the large number of studies published to date, there is only limited evidence of the beneficial effect of adherence to traditional MedDiet in maintaining a healthy body weight in childhood [25]. Moreover, with scientific evidence coming from mostly cross-sectional studies, it is difficult to draw conclusions on the effects of MedDiet adherence for improving anthropometric indicators among youth [25]. To our knowledge, no systematic review with meta-analysis has separately synthesized the effects of MedDiet-based interventions in children and adolescents in relation to the effects on anthropometric measures. A better understanding of the effects of MedDiet-based interventions on anthropometric variables could facilitate their implementation in efforts to reduce obesity in the young population. Therefore, the aim of the present meta-analysis was to evaluate the effects of MedDiet-based interventions on anthropometric and obesity indicators among children and adolescents.

## Methods

### Search strategy and selection of studies

The present review and meta-analysis were reported in accordance with the principles of the PRISMA statement [26] and following the recommendations of the Cochrane Collaboration Manual for Systematic Reviews of Interventions [27]. This systematic review and meta-analysis were registered at PROSPERO (registry number CRD42023386789).

### Eligibility criteria

Eligible articles were randomized controlled trials (RCTs) measuring the effect of an intervention based on the promotion of MedDiet and obesity-associated parameters. The search and selection of studies were performed by 2 independent reviewers who examined the titles and abstracts. Inclusion criteria were as follows: *1*) children or adolescents ≤18 y old; *2*) outcomes, assessments of anthropometric indicators using standardized tests; *3*) study design: RCTs; and *4*) studies with MedDiet-based interventions. Studies were excluded if they were review articles, editorials, or case reports.

### Information sources and search strategy

A systematic search of the MEDLINE (via PubMed), Scopus, Cochrane, and Web of Science databases included all studies up until 15 March, 2023. The search strategy included the following terms: *1*) “children,” “adolescents,” “youths,” or “preschoolers,” “teenagers”; *2*) “Mediterranean diet”; *3*) “anthropometric measurements,” “body mass index,” “obesity,” “overweight,” “excess weight,” “adiposity,” “abdominal obesity,” “body fat,” “fat mass,” or “high trunk fat mass”; and *4*) “intervention,” “clinical trial,” “randomized clinical trial,” “randomized clinical trial,” “randomized controlled trial,” “randomized controlled trial,” or “RCT.” Finally, the reference lists of the studies included in this systematic review were examined to find any relevant studies. Supplemental Table 1 shows the complete MEDLINE search strategy.

### The selection process

The strategy was designed around the Participants, Intervention, Comparison, Outcome, and Study design (PICOS) question format: Are there differences in children and adolescents (participants) who participate in an MedDiet-based intervention (intervention) compared with those who do not participate (comparator) in anthropometric or obesity indicators (BMI, waist circumference [WC], waist-to-height ratio [WHtR], percentage of obesity, or percentage of abdominal obesity) (outcome) in RCTs (study design)?

### Data collection process and data items

The following information was extracted from the included studies: *1*) reference (y); *2*) country of intervention; *3*) characteristics of subjects, including total sample size, size of intervention and control group, sex ratio, age range, mean age, and baseline weight status; *4*) duration of intervention (wk); *5*) type of intervention; and *6*) outcome information, including information on the anthropometric indicators and obesity prevalence analyzed. All results compatible with each outcome domain in each study were included. In the case of missing information in the published article, the corresponding authors of these studies were contacted directly to request the information.

### Study risk of bias assessment

The risk of bias of the RCTs was assessed using the Cochrane risk of bias tool for randomized trials or cluster randomized trials (RoB 2.0) [28], in which 5 domains were assessed: randomization process, deviations from intended interventions, missing outcome data, outcome measurement, and selection of the reported outcome. The risk of bias was assessed in each domain. Studies were classified as *1*) “low risk” of bias when a low risk of bias was determined for all domains; *2*) “some concerns” if ≥1 domain was assessed as raising some concerns but did not have a high risk of bias for any individual domain; or *3*) “high risk” of bias when a high risk of bias was obtained for ≥1 domain or the study judgment included “some concerns” across multiple domains [28].

### Effect measures

The effect size of each study was estimated by Cohen’s *d* and included in the meta-analysis to verify the effect of MedDiet-based interventions on continuous markers related to childhood obesity (BMI, WC, and WHtR). For categorical markers (that is, percentage of obesity and abdominal obesity), the risk difference and the number needed to treat (NNT) were determined [27]. The risk difference is the difference between the observed risks (proportions of individuals with the outcome of interest) in the 2 groups. The NNT was applied, being the expected number of people who need to receive the experimental rather than the comparator intervention for one additional person to either incur or avoid an event in a given time frame. Analyses were performed using Stata 17.0 software (Stata, College Station, TX, United States) for Windows.

### Synthesis methods

Different meta-analyses were independently performed to examine the effect of MedDiet promotion-based interventions on anthropometric and weight management indicators, such as BMI, WC, WHtR, percentage of obesity, and percentage of abdominal obesity. We used a common interpretation to report effect sizes as small (*d* ∼ 0.2), medium (*d* ∼ 0.5), and large (*d* ∼ 0.8) [29]. For both, the Hartung–Knapp–Sidik–Jonkman method for random effects was used to calculate the overall estimate of the effect size and its respective 95% CIs. This decision is justified by the fact that the Hartung–Knapp–Sidik–Jonkman method consistently provides better error rates than the traditional DerSimonian and Laird method, especially when the number of studies is small [30]. Furthermore, prior to analyses, multiple groups from Lisón et al. [31] were combined groups to create a single pairwise comparison (as recommended by the Cochrane Collaboration Manual for Systematic Reviews of Interventions [27]). Heterogeneity was examined using the *I*^2^ statistic, which ranges from 0% to 100% [32]. The *I*^2^ values were interpreted as not important (0% to 30%), moderate (30% to 60%), substantial (60% to 75%), or considerable (75% to 100%).

To assess the robustness of the estimates obtained, sensitivity analyses were performed by eliminating (one at a time) each study from the total estimates. In addition, subgroup analyses were performed according to the type of country in which the intervention was carried out (that is, Mediterranean or nonMediterranean country), as well as according to the baseline weight status of the participants (that is, all weight statuses or only participants with overweight/obesity). Meta-regression analyses were also performed to address whether the duration of the intervention or the mean age of participants could modify the effect of MedDiet-based interventions on the weight management indicators analyzed.

### Reporting bias assessment

Small study effects and publication biases were examined using the Doi plot and the Luis Furuya-Kanamori (LFK) index [33]. No asymmetry, minor asymmetry, or major asymmetry were assigned values of 1, between 1 and 2, and 2, respectively [33].

## Results

### Study selection

**FIGURE 1:**
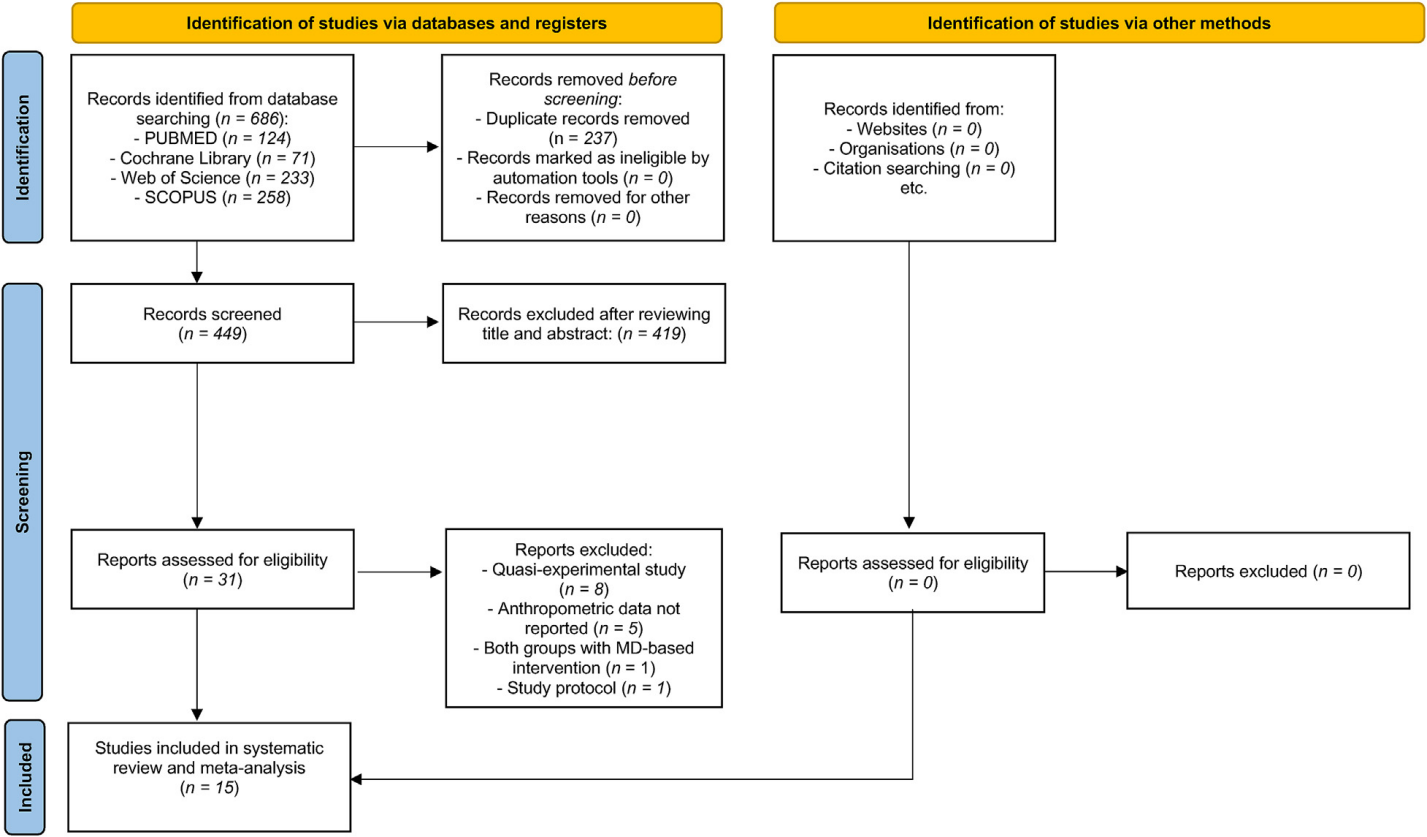
PRISMA 2020 flow chart for systematic reviews that included database searches. MedDiet, Mediterranean diet.

A total of 15 RCTs were included in the meta-analysis [31,[34], [35], [36], [37], [38], [39], [40], [41], [42], [43], [44], [45], [46], [47]]. The PRISMA flow chart is shown in Figure 1. Excluded studies and reasons for exclusion are provided in Supplemental Table 2.

### Study characteristics

Table 1 includes a summary of the 15 RCTs included in this study. All children and adolescents were recruited from educational centers (schools, high schools) or hospitals. The RCTs included a total of 7184 participants (intervention groups: *n* = 3356) aged between 3 [35,[38], [39], [40]] and 18 [39,44,47] y. Fourteen studies included participants of both sexes, with the exception of one study that included only girls. The duration of the MedDiet-based interventions was ≥8 wk. A summary of the type of actions carried out in both the intervention and control groups can be found in Supplemental Table 3. Similarly, detailed information about the RCTs is presented in Supplemental Table 4.

**TABLE 1:** A summary of the characteristics of the included studies (*n* = 15)

Authors	Country	Age group	Sample	Weight status (baseline)	Duration (wk)	Intervention (type)	Indicators examined
Lisón et al. [31]	Spain	6–16	*n* = 110	Excess weight §	36	The prescribed diet and physical exercise	BMI, WC, WHtR, %OB, %AO
			IG a = 45				
			IG b = 41				
			CG = 24				
Velázquez-López et al. [39]	Mexico	3–18	*n* = 49	Excess weight §	16	The prescribed diet	BMI, WC, WHtR, %OB, %AO
			IG = 24				
			CG = 25				
Muros et al. [45]	Spain	10–11	*n* = 135	All group †	24	Nutritional education	BMI, WC, %OB
			IG c = 21				
			CG = 41				
Peñalvo et al. [40]	Spain	3–5	*n* = 1779	All group †	52–156	Nutritional education and physical activity	BMI, WC, WHtR, %OB, %AO
			IG = 948				
			CG = 831				
Akdemir et al. [34]	Turkey	6–14	*n* = 1288	All group †	32	Nutritional education	BMI, %OB
			IG = 647				
			CG = 641				
Bibiloni et al. [35]	Spain	3–7	*n* = 1119	All group †	48	Nutritional education	BMI, WHtR, %OB, %AO
			IG = 319				
			CG = 880				
Gómez et al. [37]	Spain	8–10	*n* = 2086	All group †	60	Nutritional education and physical activity	BMI, WC, WHtR, %OB, %AO
			IG = 974				
			CG = 1112				
Ojeda-Rodríguez et al. [41]	Spain	7–16	*n* = 107	Excess weight §	8	The prescribed diet and physical activity	BMI, WC, WHtR, %OB, %AO
			IG = 81				
			CG = 26				
Akbulut et al. [43]	Turkey	9–17	*n* = 45	Excess weight §	12	The prescribed diet and physical exercise	BMI
			IG = 23				
			CG = 22				
Fernández-Ruiz et al. [36]	Spain	6–12	*n* = 101	Excess weight §	40	Nutritional education and physical exercise	BMI, WC, WHtR, %OB, %AO
			IG = 51				
			CG = 50				
Prieto-Zambrano et al. [42]	Spain	11–15	*n* = 82	All group †	20	Nutritional education	BMI, WC, WHtR, %OB, %AO
			IG = 46				
			CG = 36				
Yurtdaş et al. [47]	Turkey	11–18	*n* = 44	Excess weight §	12	The prescribed diet	BMI, WC, WHtR
			IG = 22				
			CG = 22				
Blancas-Sánchez et al. [46]	Spain	9–15	*n* = 29	All group †	20	Nutritional education	BMI, WC, WHtR, %OB, %AO
			IG = 14				
			CG = 15				
Martíncrespo-Blanco et al. [38]	Spain	3–5	*n* = 133	All group †	36	Nutritional education	BMI, %OB
			IG = 65				
			CG = 68				
Asoudeh et al. [44]	Iran	13–18	*n* = 70	Excess weight §	12	The prescribed diet	BMI, WC, WHtR
			IG = 35				
			CG = 35				

AO, abdominal obesity; BMI, body mass index; CG, control group; IG, intervention group; OB, obesity; WC, waist circumference; WHtR, waist-to-height ratio.

a Intervention carried out in hospital.

b Intervention carried out at home.

c Group that only received nutritional education.

† Participants with any weight status (thinness, normal weight, overweight, or obesity).

§ Participants with excess weight (overweight or obesity).

### Risk of bias in studies

Fifteen RCTs were evaluated according to the RoB 2.0 tool [28], of which 6 were rated as “low risk” of bias [36,38,40,43,46,47], 8 as “some concerns” of bias [31,34,35,39,41,42,44,45], and one as “high risk” [37] (Supplemental Figure 1).

### Summary of the results

**FIGURE 2:**
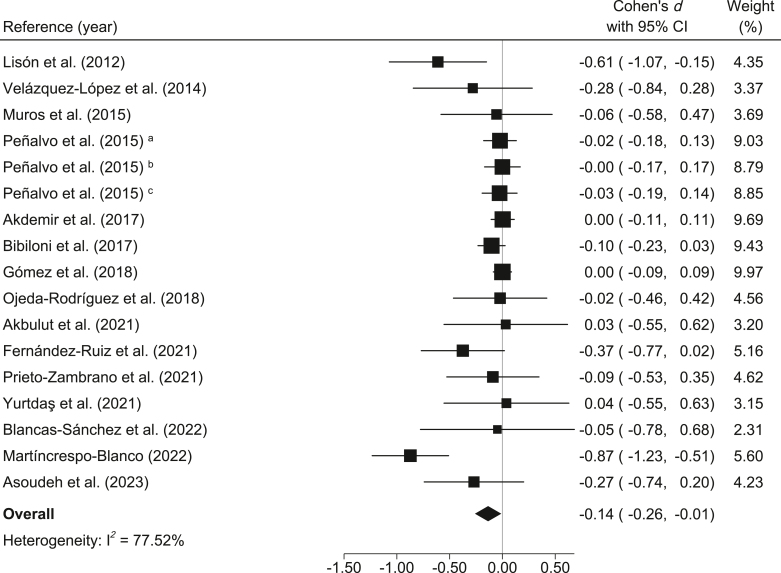
Meta-analysis of Mediterranean diet-based interventions to determine the combined effect size on BMI. ^a^3-y age group; ^b^4-y age group; ^c^5-y age group.

BMI: Compared to the control group, the MedDiet-based interventions showed small and significant reductions in BMI (*d* = −0.14; 95% CI: −0.26, −0.01; *I*^2^ = 77.52%) (Figure 2). Furthermore, the reduction in absolute BMI was −0.35 kg/m^2^ (95% CI: −0.59, −0.04; *I*^2^ = 75.00%).

**FIGURE 3:**
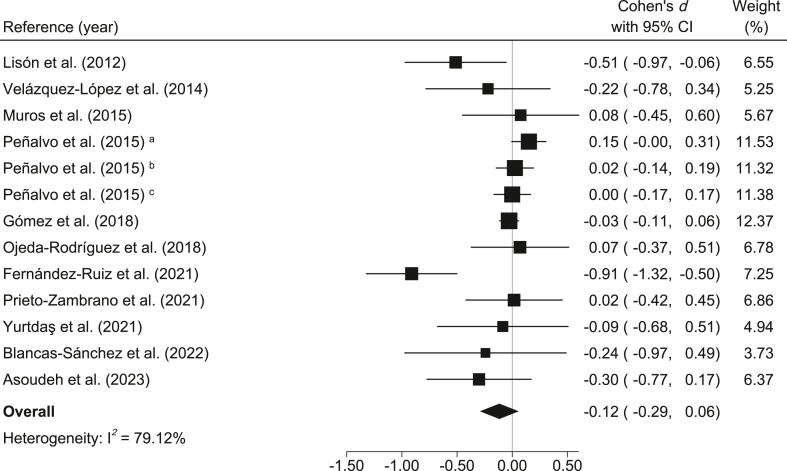
Meta-analysis of Mediterranean diet-based interventions to determine the combined effect size on the waist circumference. ^a^3-y age group; ^b^4-y age group; ^c^5-y age group.

WC: When comparing the control groups with the MedDiet-based intervention groups, small and nonsignificant decreases in WC were found (*d* = −0.12; 95% CI: −0.29, 0.06; *I*^2^ = 79.12%) (Figure 3). In addition, the reduction in absolute WC was −0.56 cm (95% CI: −1.41, 0.29; *I*^2^ = 76.68%).

**FIGURE 4:**
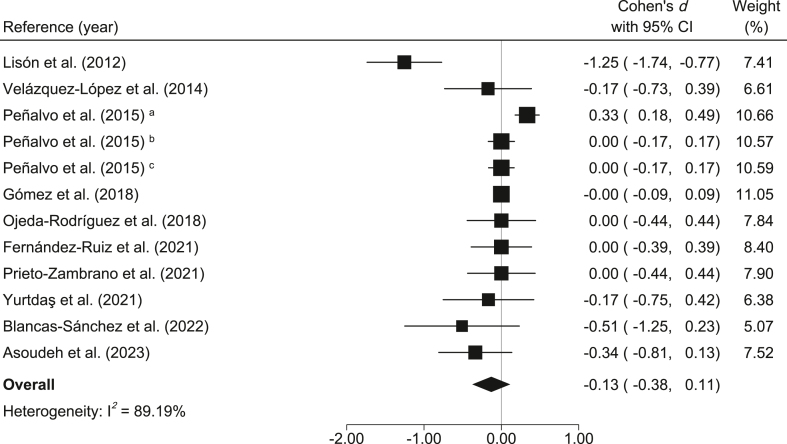
Meta-analysis of Mediterranean diet-based interventions to determine the combined effect size on waist-to-height ratio. ^a^3-y age group; ^b^4-y age group; ^c^5-y age group.

WHtR: Compared to the control group, the MedDiet-based interventions showed small and nonsignificant reductions in WHtR (*d* = −0.13; 95% CI: −0.38, 0.11; *I*^2^ = 89.19%) (Figure 4). Moreover, the reduction in absolute WHtR was −0.001 (95% CI: −0.006, 0.004; *I*^2^ = 86.43%).

**FIGURE 5:**
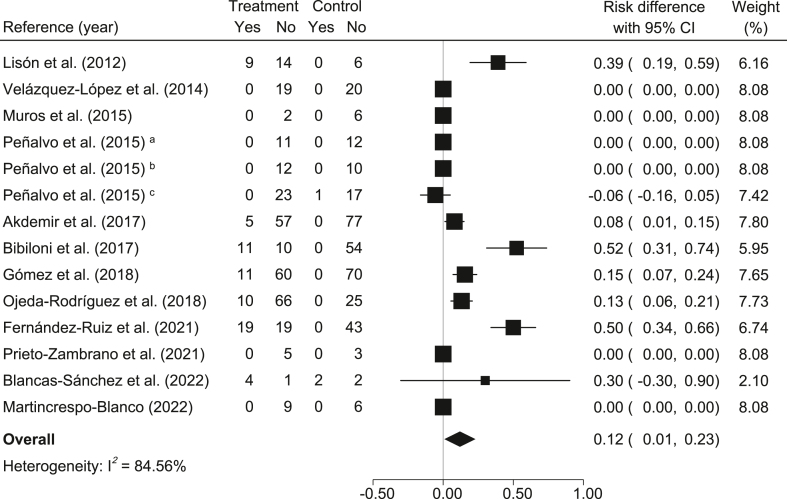
Meta-analysis of Mediterranean diet-based interventions to determine the combined effect size on the relative risk difference for obesity percentage. ^a^3-y age group; ^b^4-y age group; ^c^5-y age group.

The percentage of obesity: In relation to the control group, participants in the MedDiet-based interventions had a significant reduction in the percentage of obesity (*risk difference* = 0.12; 95% CI: 0.01, 0.23; *I*^2^ = 84.56%) (Figure 5). Furthermore, the NNT for one participant to benefit was ∼7 (100% / 12% ≃ 6.67).

**FIGURE 6:**
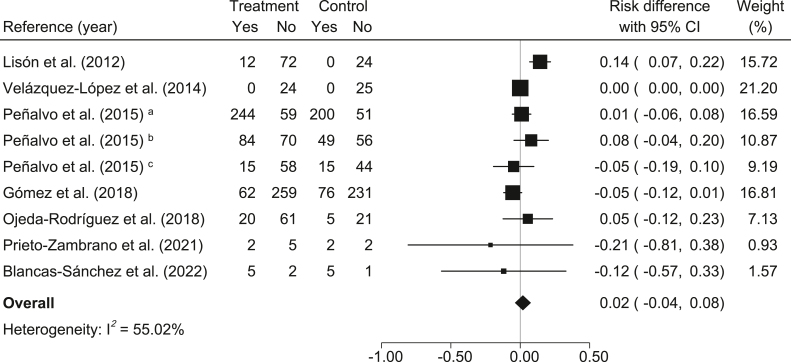
Meta-analysis of Mediterranean diet-based interventions to determine the combined effect size on the relative risk difference for abdominal obesity percentage. ^a^3-y age group; ^b^4-y age group; ^c^5-y age group.

The percentage of abdominal obesity: In relation to the control group, participants in the MedDiet-based interventions had a nonsignificant reduction in the percentage of abdominal obesity (*risk difference* = 0.02; 95% CI: −0.04, 0.08; *I*^2^ = 55.02%) (Figure 6). In addition, the NNT for one participant to benefit was 50 (100% / 2% = 50).

### Sensitivity analysis, subgroup analysis, and meta-regression

After removing the studies from the analyses individually by sensitivity analysis, none of the RCTs substantially changed the estimate of effects (BMI, percentage of obesity) in the intervention group versus the control group (*p* < 0.10 for all cases) (Supplemental Figures 2–6).

In relation to subgroup analyses, the effects of the interventions were verified according to the regions where the interventions were conducted (Mediterranean or nonMediterranean) or the weight status of the participants (all weight statuses or only participants with overweight/obesity) (Table 2). A further subgroup analysis according to the type of intervention (that is, prescribed diet, nutritional education, or prescribed diet or nutritional education and physical activity or exercise) is shown in Supplemental Table 5. Concerning the type of country where the intervention was conducted, significant reductions were found in interventions performed in Mediterranean countries (that is, Spain and Turkey) for BMI (risk difference = 0.13; 95% CI: 0.01, 0.25; *I*^2^ = 86.15%) but were not significant for interventions performed in a nonMediterranean country (that is, Mexico). In addition, subgroup analysis by weight status showed that the interventions had greater effects when aiming at participants with excess weight for BMI, WC, WHtR, percentage of obesity, and percentage of abdominal obesity. However, statistically significant differences were only found for WC (*p* = 0.030). The NNT for one participant to reduce obesity was ∼4 (100% / 24% ≃ 4.17).

**TABLE 2:** Subgroup analyses according to the type of country or baseline weight status

Variables	#	*d*	LLCI	ULCI	*I*^2^	*p*
Country						
BMI						
MedDiet countries	13	−0.13	−0.27	0.01	81.54	0.45
Non-MedDiet countries	2	−0.27	−0.34	−0.20	0.00	
WC						
MedDiet countries	9	−0.10	−0.31	0.11	83.94	0.43
Non-MedDiet countries	2	−0.27	−0.78	0.24	0.11	
WHtR						
MedDiet countries	9	−0.12	−0.42	0.18	92.25	0.51
Non-MedDiet countries	2	−0.27	−1.30	0.76	1.68	
% Obesity						
MedDiet countries	11	0.13	0.01	0.25	86.15	1.00
Non-MedDiet country	1	0.00	0.00	0.00	—	
% Abdominal obesity						
MedDiet countries	6	0.02	−0.05	0.10	62.21	1.00
Non-MedDiet country	1	0.00	0.00	0.00	—	
Weight status (baseline)						
BMI						
All group †	8	−0.11	−0.29	0.06	87.52	0.48
Excess weight §	7	−0.22	−0.45	0.02	28.64	
WC						
All group †	5	0.02	−0.07	0.11	42.25	0.03
Excess weight §	6	−0.34	−0.74	0.05	54.62	
WHtR						
All group †	4	0.04	−0.16	0.24	77.88	0.08
Excess weight §	6	−0.34	−0.86	0.18	72.31	
% Obesity						
All group †	8	0.06	−0.06	0.18	77.90	0.10
Excess weight §	4	0.24	−0.03	0.51	78.60	
% Abdominal obesity						
All group †	4	−0.01	−0.09	0.07	53.21	0.28
Excess weight §	3	0.05	−0.08	0.19	27.19	

BMI, body mass index; MedDiet, Mediterranean diet; LLCI, lower limit confidence interval; ULCI, upper limit confidence interval; WC, waist circumference; WHtR, waist-to-height ratio. #, number of studies examined.

† Participants with any weight status (thinness, normal weight, overweight, or obesity).

§ Participants with excess weight (overweight or obesity).

Random-effects meta-regression models are shown in Table 3. Both intervention duration and age mean were not statistically significant in terms of effect sizes (BMI, WC, WHtR, percentage of obesity, and percentage of abdominal obesity) (Table 3).

**TABLE 3:** Meta-regression analyses by intervention duration and mean age

Variables	*B*	SE	LLCI	ULCI	*p*
BMI					
Duration (wk)	0.001	0.001	−0.002	0.004	0.388
Mean age (y)	0.004	0.017	−0.033	0.041	0.833
WC					
Duration (wk)	0.002	0.002	−0.001	0.006	0.203
Mean age (y)	−0.026	0.022	−0.075	0.023	0.270
WHtR					
Duration (wk)	0.004	0.002	−0.001	0.009	0.123
Mean age (y)	−0.048	0.029	−0.112	0.016	0.128
% Obesity					
Duration (wk)	−0.001	0.001	−0.004	0.002	0.584
Mean age (y)	0.006	0.017	−0.031	0.042	0.732
% Abdominal obesity					
Duration (wk)	−0.001	0.001	−0.001	0.001	0.972
Mean age (y)	−0.001	0.008	−0.019	0.019	0.975

LLCI, lower limit confidence interval; SE, standard error; ULCI, upper limit confidence interval; WC, waist circumference; WHtR, waist-to-height ratio; *B*, unstandardized beta coefficient.

### Reporting biases

A major asymmetry suggestive of small study effects was observed for BMI (LFK index = −3.36) (Supplemental Figure 7), WC (LFK index = −2.48) (Supplemental Figure 8), WHtR (LFK index = −2.13) (Supplemental Figure 9), percentage of obesity (LFK index = 3.47) (Supplemental Figure 10), and percentage of abdominal obesity (LFK index = −2.21) (Supplemental Figure 11).

## Discussion

To our knowledge, this is the first systematic review with meta-analysis that separately synthesized the effects of MedDiet-based interventions in children and adolescents in relation to the effects on anthropometric measures. Overall, we found that MedDiet-based interventions (15 RCTs with a minimum duration of 8 wk) decreased BMI and the percentage of obesity. Our analyses indicate that for every 7 young people treated with MedDiet-based interventions, one would no longer have obesity. Additionally, such interventions had greater effects in reducing obesity when conducted in Mediterranean countries (that is, Spain and Turkey) and when they included only young people with excess weight. Because heterogeneity for most outcomes was substantial to considerable, the results should be interpreted with caution. There are certain factors that could explain this high heterogeneity. For example, the fact that the interventions included diet-based interventions, nutrition education, or a combination of diet plus physical activity or exercise could explain this inconsistency between studies. Likewise, having interventions in Mediterranean and nonMediterranean countries, the different age groups included (that is, preschoolers, children, adolescents), or performing the interventions only on young people or including parents/families could be other reasons justifying this aspect.

Previous systematic reviews have shown inconclusive results in relation to adherence to MedDiet and obesity-related markers among children/adolescents [25,48]. However, none of these reviews provided a meta-analysis of RCTs, which could explain the discrepancy. In this line, Iaccarino Idelson et al. [48] found mixed results on the association between MedDiet and anthropometric indicators or body composition in a previous systematic review of children and adolescents. In that review, only 10 of 26 papers reported that higher adherence to MedDiet was associated with lower BMI values or prevalence of excess weight. Furthermore, these authors pointed out that the results are even less consistent (due to the lack of studies) when analyzing the relationship between MedDiet and abdominal obesity (through WC) or level of adiposity. A possible explanation for this discrepancy may be due to the design of the studies analyzed in this systematic review since most of them were observational (that is, cross-sectional, longitudinal), in which it is not possible to infer cause-effect relationships. Conversely, Lassale et al. [25] concluded in a recent systematic review that, despite the large number of published studies, there is only limited evidence of the beneficial effect of following a traditional MedDiet in maintaining a healthy body weight in childhood. However, these same authors only conducted their systematic review using the MEDLINE database, excluding several studies that could have affected the results obtained.

There are several plausible mechanisms by which following an MedDiet can help maintain a healthy body weight and prevent obesity early in life. First, one of the basic tenets of MedDiet is the intake of plant-based foods, such as fruits and vegetables, which are characterized by high volumes with low energy density. Thus, high-volume foods may require more time to be ingested than low-volume foods, and prolonging the duration of the meal may increase satiety and reduce energy intake [49]. In addition, some essential components of the MedDiet (that is, fruits, vegetables, whole grains, nuts, or seeds), which are high fiber-rich, nutrient-dense, energy-poor foods [50], as well as family meals [51] (characteristic of the MedDiet), may promote consuming fewer calories. Moreover, specific compounds such as olive oil phenolic compounds, omega-3 polyunsaturated fatty acids, vitamins, trace elements, and polyphenols are found in abundance in a traditional MedDiet [17]. They have been shown to modulate and maintain healthy gut microbiota, as well as improve gut barrier integrity, which has been shown to be altered in the presence of obesity and metabolic syndrome [52].

On the other hand, the intake of ultra-processed foods (away from the basic principles of MedDiet) has been associated with an increased dietary risk of associated noncommunicable diseases (for example, excess weight), as they are high in calories and low in nutrients, and may contribute to a higher caloric intake [53]. Likewise, a dietary pattern characterized by a diet based on energy-dense, low-fiber ultra-processed foods at the age of 3 y is associated with excess weight and elevated BMI later in childhood [54]. In addition, a diet high in ultra-processed foods (for example, snacks, cookies), which are usually rich in added sugars, salt, or saturated fats, is consumed significantly faster than an unprocessed diet (for example, fruits, vegetables), which may contribute to higher caloric intake [55].

Concerning subgroup analysis according to the type of country where the intervention was conducted, we found that the interventions had greater effects in reducing obesity when conducted in Mediterranean countries (that is, Spain, Turkey), than they were when conducted in a nonMediterranean country (that is, Mexico). However, these results should be interpreted with some caution when ruling out this type of intervention in nonMediterranean countries. First, with respect to nonMediterranean countries, only a single study examining the reduction of obesity with complete data was included [39]. Further studies are required to verify whether MedDiet-based interventions work in nonMediterranean countries. Notwithstanding, some MedDiet-based interventions performed in nonMediterranean countries have shown beneficial effects on health outcomes [56,57]. Second, we observed a quantitative interaction since the size of the effect for nonMediterranean country interventions varied but not the direction [58]. Third, despite the traditional definition of MedDiet as the typical dietary habit of individuals living in countries surrounding the Mediterranean Sea, other Mediterranean-type ecosystems can be located from the 30^th^ parallel to the 45^th^ parallel of the south or north latitude with their coasts facing west (for example, central Chile). In addition, agriculture produced at some of these latitudes offers food similar to that of Mediterranean countries [59] due to their latitude, climate, biodiversity, and agriculture being similar to the Mediterranean-type ecosystem.

In relation to the baseline weight status of the participants, interventions had higher effects when the targets were young participants with excess weight than when they included all weight statuses (that is, underweight, normal weight, overweight, or obesity). This result could be a clinically relevant finding since it seems to suggest that MedDiet-based interventions could be a useful strategy for the treatment of obesity in the young population. A possible explanation for this discrepancy could lie in the fact that by including young people with normal weight and possibly increasing the number of participants, the interventions may have lower effects. Furthermore, the relationship between anthropometric or obesity-related markers and health is not linear [1]. Decreases in BMI for young people with normal weight could change their weight status toward underweight, which would not lead to health benefits. Another possible reason is that young people with excess weight have higher baseline BMI levels and, therefore, a greater margin of benefit from interventions. In addition, caution is necessary when interpreting the results of studies that included participants with all weight statuses. It is possible that by segmenting the results according to weight status, the results would have reported greater effects in the young population with excess weight.

The interpretation of these results should be viewed with caution as they have some limitations. First, the main limitation of this review is the low quality of the included studies. Second, only a few RCTs considered important confounding factors such as physical activity, sedentary behavior, or sleep duration. Third, not all interventions were exclusively based on MedDiet content. Some of them also incorporated physical activity promotion or physical exercise (among other components), which could have overestimated the pooled effects obtained. However, given that some groups included physical activity or exercise in the control and intervention groups, our results seem to suggest that MedDiet could offer additional benefits to the effect of physical activity or exercise on anthropometric and obesity markers in this population. Fourth, not all RCTs reported compliance with intervention sessions (for example, number of sessions attended), and it is essential to know whether participants acted as planned and adhered to the planned program. Fifth, not all RCTs reported on prior knowledge/adherence to the MedDiet. Sixth, the use of BMI z score could be more appropriate as an anthropometric indicator than crude BMI. However, we were unable to perform an analysis of the BMI z score due to the low number of studies that reported it and the use of different criteria (that is, WHO, International Obesity Task Force). Nonetheless, the percentage of obesity in all studies was adjusted for age and sex, and significant effects of the MedDiet on reducing obesity were also observed. Finally, publication bias may have affected the results of this review, as the Doi plot and LFK index showed larger asymmetries for all the indicators examined.

To conclude, MedDiet-based interventions have a significant effect on reducing BMI as well as reducing the risk of obesity (percentage of obesity) in children and adolescents (aged 3–18 y). The findings suggest that interventions conducted in young people with excess weight could have greater effects in improving these parameters. Furthermore, as no episodes of serious adverse effects were reported during the interventions in any of the RCTs included in the present meta-analysis, these results suggest that MedDiet-based interventions in the trial setting can be safely conducted in children and adolescents. This finding could be clinically relevant since it highlights the efficacy of MedDiet-based interventions as a useful tool in the interest of reversing the high prevalence of obesity.

## Acknowledgments

JFL-G is a Margarita Salas Fellow (Universidad Pública de Navarra–1225/2022). AG-H is a Miguel Servet Fellow (Instituto de Salud Carlos III–CP18/0150).

### Author contributions

The authors’ responsibilities were as follows—JFL-G: designed the study; JFL-G, and AG-H: contributed to the interpretation and analysis of the data; JFL-G wrote the initial draft; AG-H, MS-P, IC-R, VM-V, and SK contributed to the revision of the manuscript; and all authors: read and approved the final manuscript.

### Conflict of interest

The authors report no conflicts of interest.

### Funding

This research did not receive any specific grant from funding agencies in the public, commercial, or not-for-profit sectors.

### Author disclosures

The authors report no conflicts of interest.

### Data availability

The data that support the findings of this study are available from the corresponding author upon reasonable request.

## Appendix A Supplementary data

Multimedia component 1 Multimedia component1
