# Supplementary figures and images for: Mediterranean Diet-Based Interventions to Improve Anthropometric and Obesity Indicators in Children and Adolescents: A Systematic Review with Meta-Analysis of Randomized Controlled Trials
Source: Adv Nutr. 2023 Apr 29;14(4):858–69. doi: 10.1016/j.advnut.2023.04.011 (PMC10334150; doi:10.1016/j.advnut.2023.04.011)

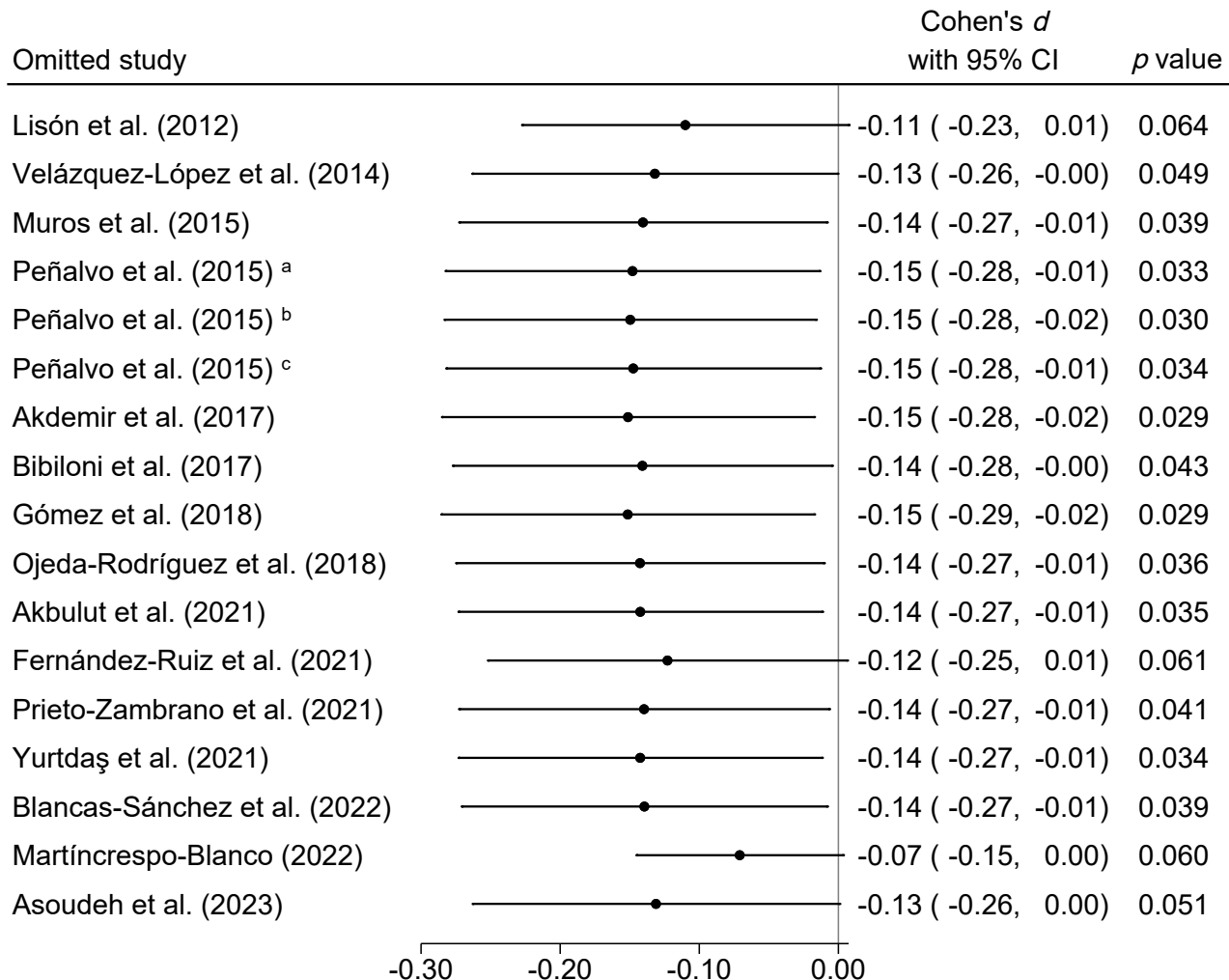

Supplement: Multimedia component 1 [file mmc1.pdf]

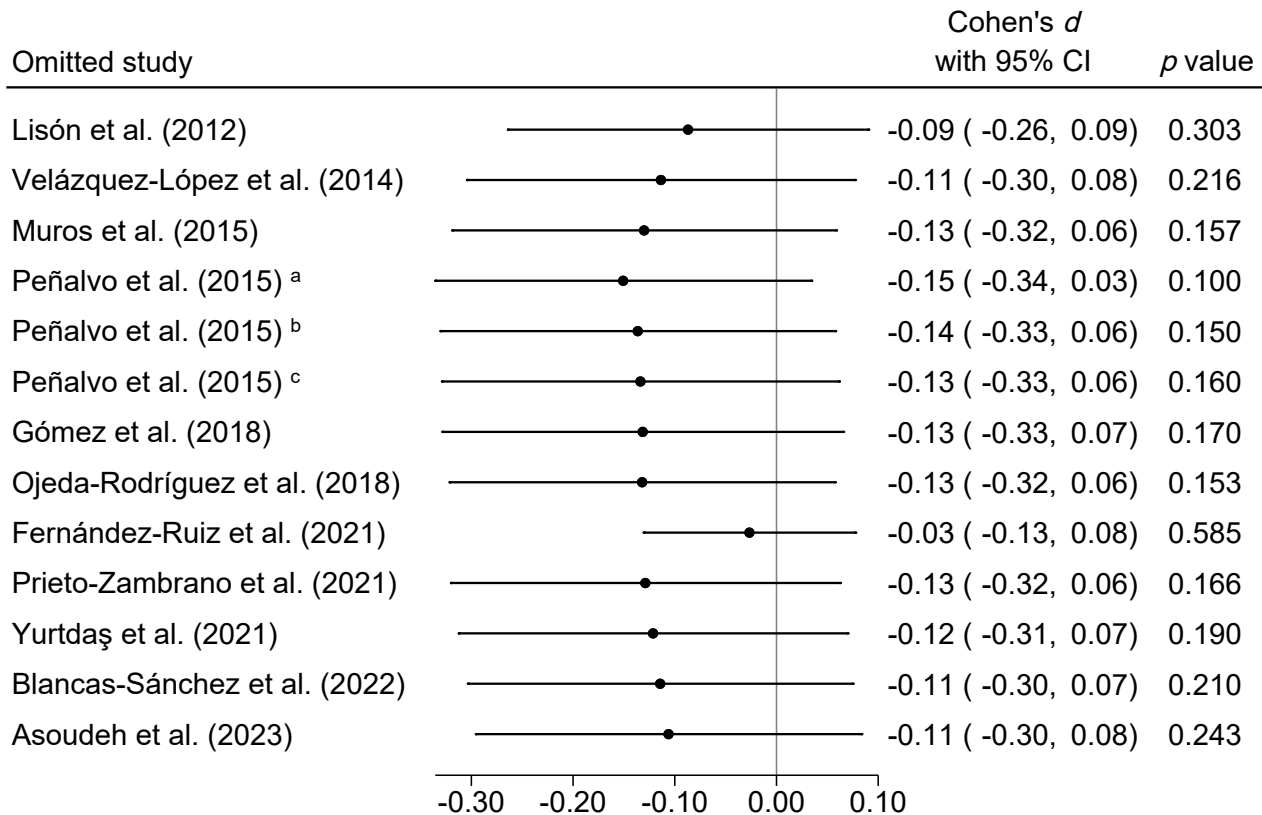

Supplement: Multimedia component 2 [file mmc2.pdf]

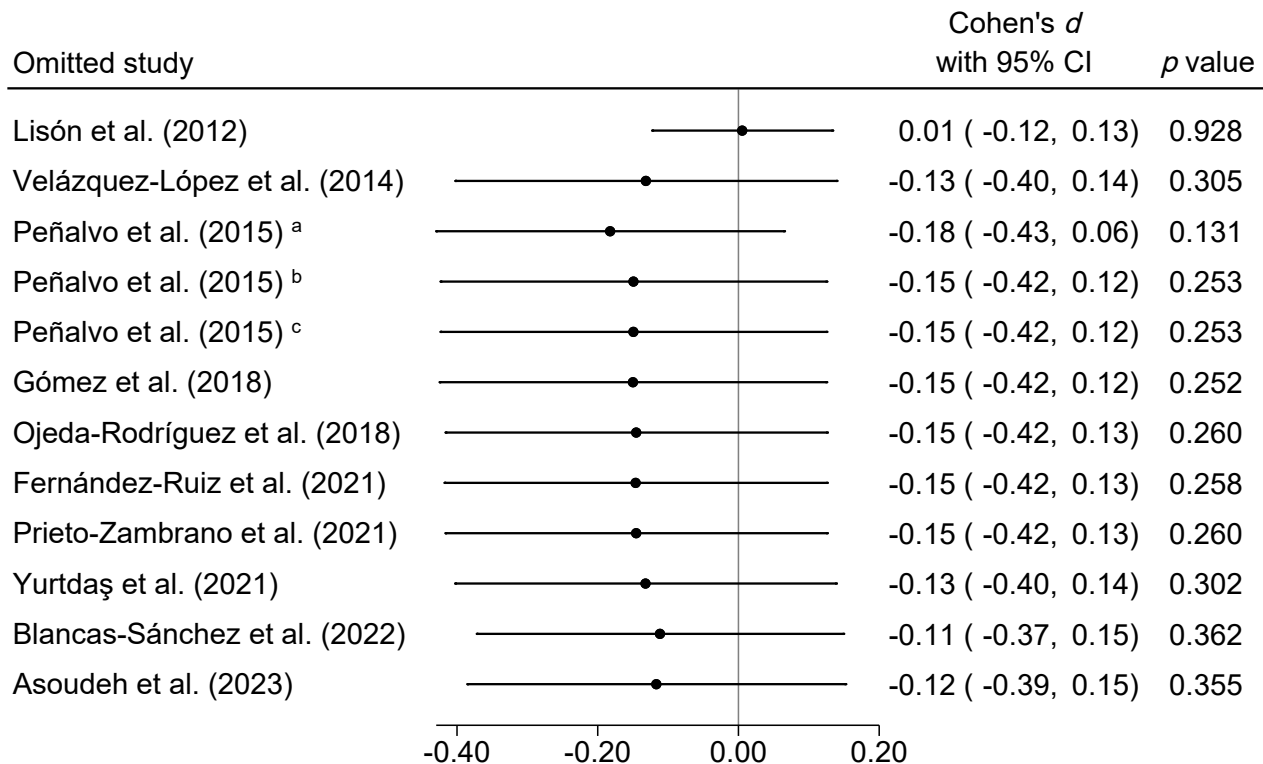

Supplement: Multimedia component 3 [file mmc3.pdf]

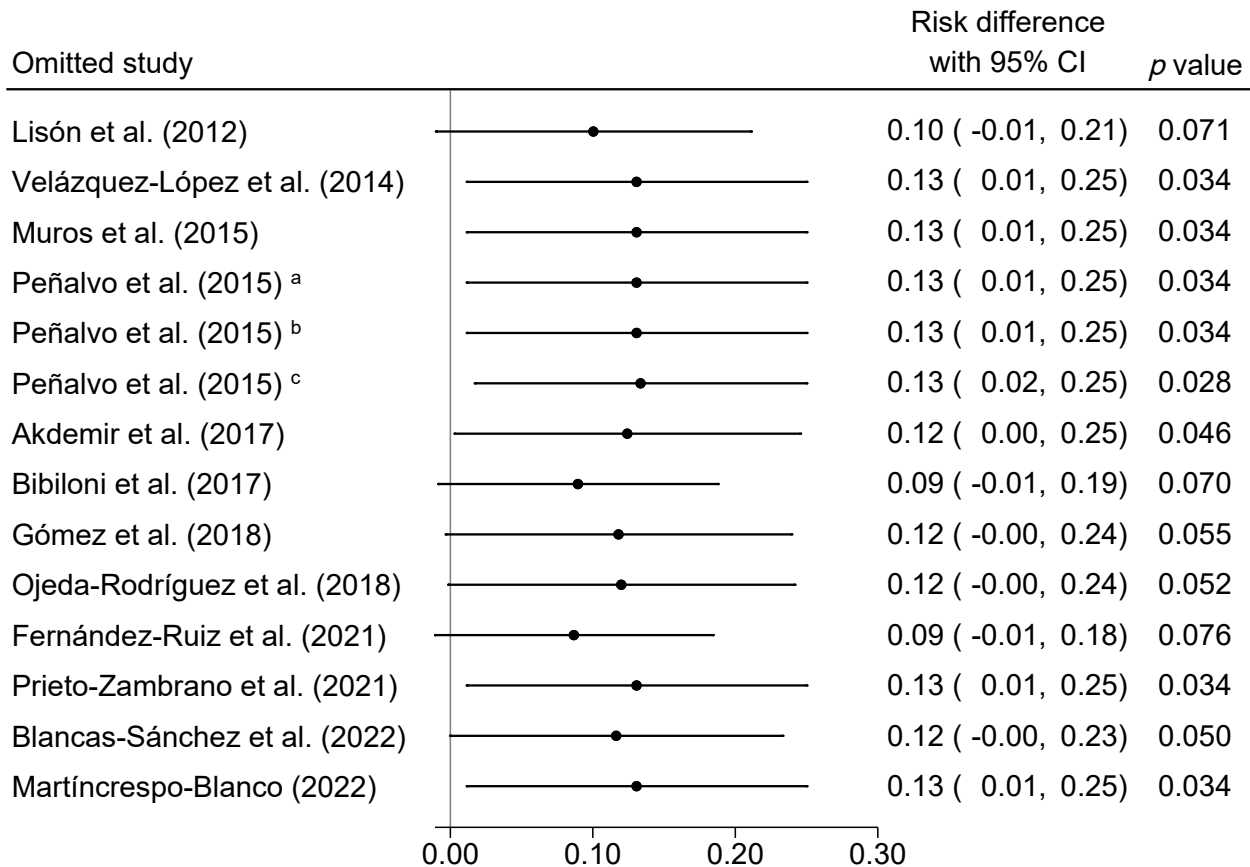

Supplement: Multimedia component 4 [file mmc4.pdf]

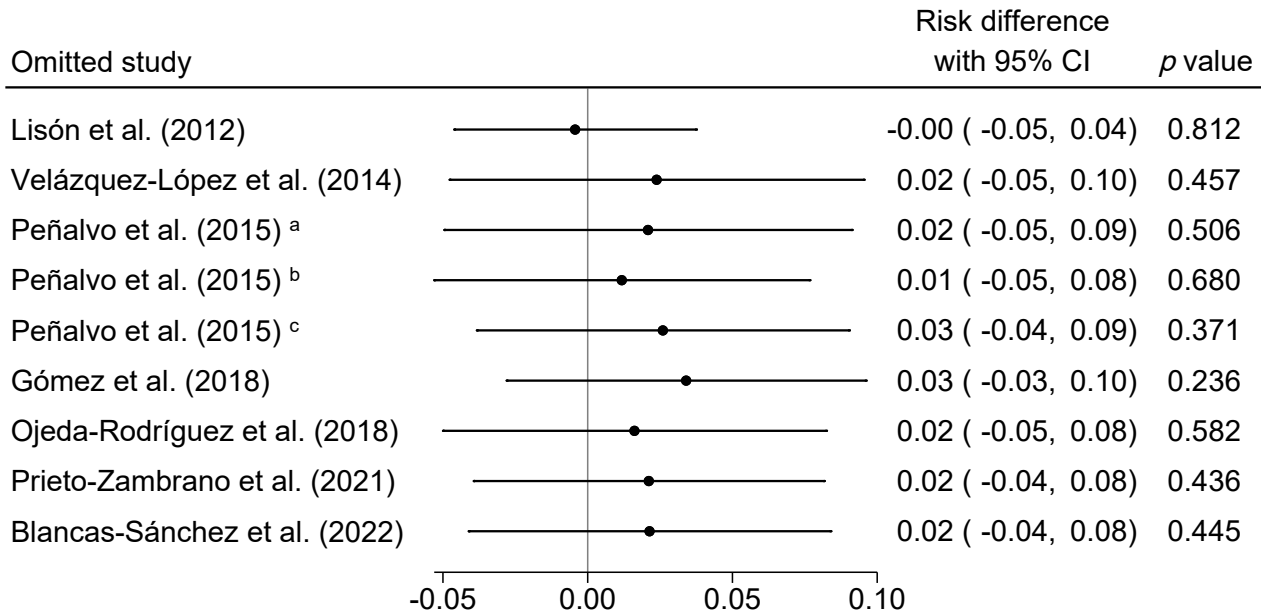

Supplement: Multimedia component 5 [file mmc5.pdf]

LFK index = -3.36 (major asymmetry)

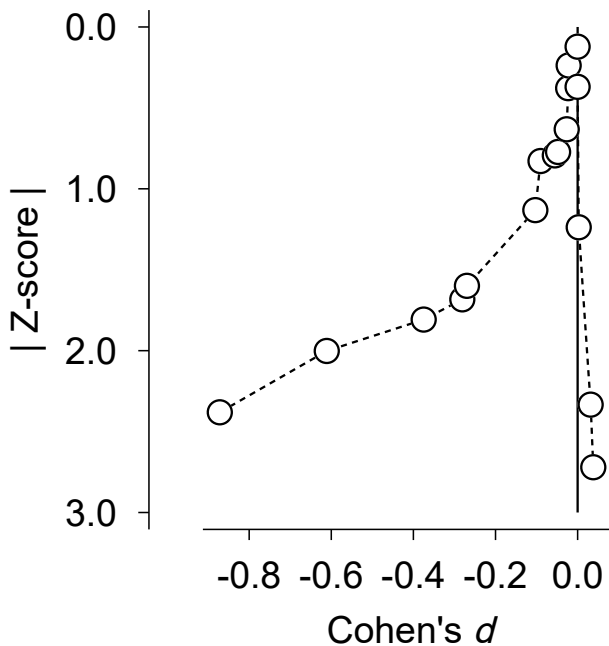

Supplement: Multimedia component 6 [file mmc6.pdf]

LFK index = -2.48 (major asymmetry)

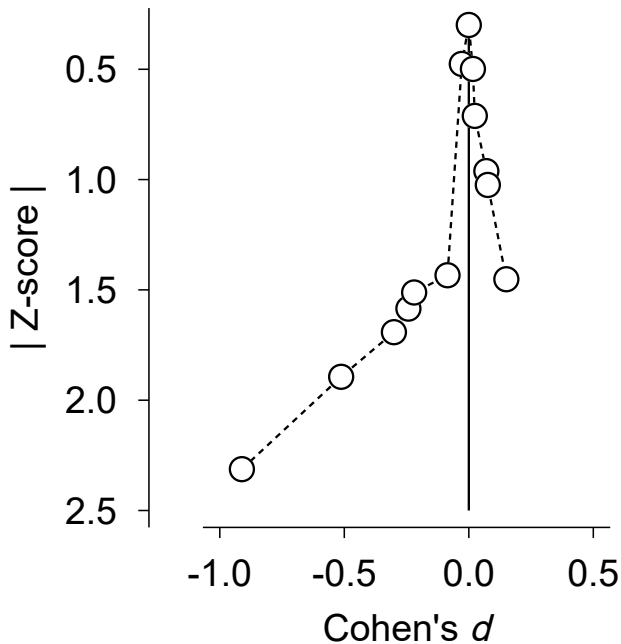

Supplement: Multimedia component 7 [file mmc7.pdf]

LFK index = -2.13 (major asymmetry)

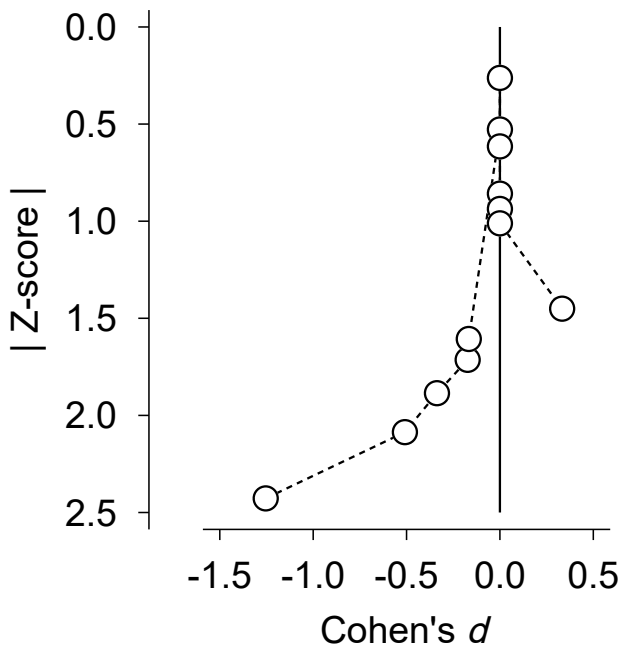

Supplement: Multimedia component 8 [file mmc8.pdf]

LFK index = 3.47 (major asymmetry)

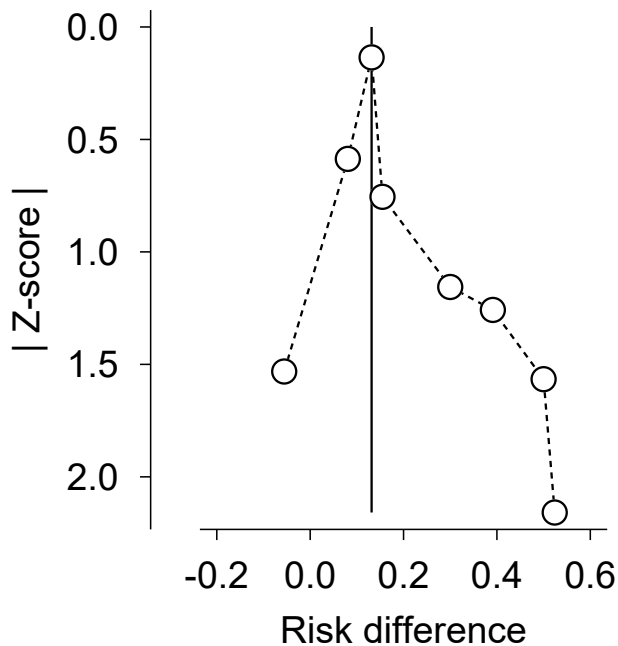

Supplement: Multimedia component 9 [file mmc9.pdf]

LFK index = -2.21 (major asymmetry)

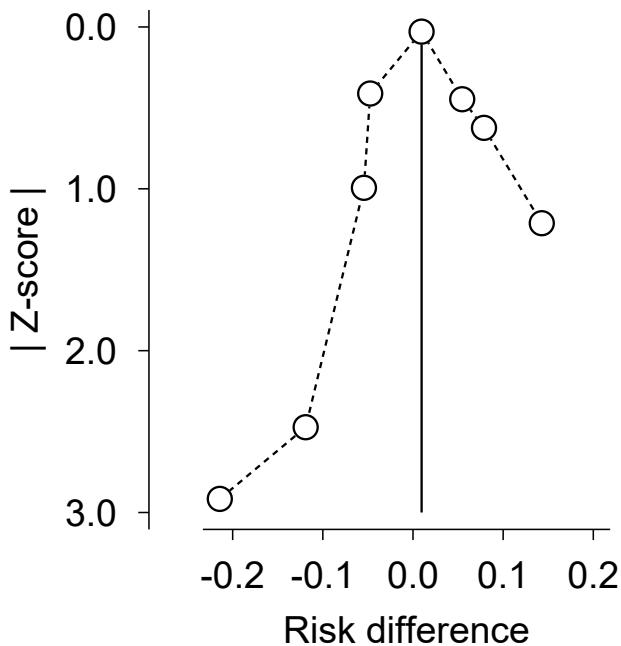

Supplement: Multimedia component 10 [file mmc10.pdf]
