# Supplementary material for: Mediterranean Diet-Based Interventions to Improve Anthropometric and Obesity Indicators in Children and Adolescents: A Systematic Review with Meta-Analysis of Randomized Controlled Trials
Source: Adv Nutr. 2023 Apr 29;14(4):858–69. doi: 10.1016/j.advnut.2023.04.011 (PMC10334150; doi:10.1016/j.advnut.2023.04.011)
Supplement: Multimedia component 11 [file mmc11.docx]

| **Participants (P)** | |  | **Intervention (I)** |  | **Outcomes (O)** |  | **Study design (S)** |
| --- | --- | --- | --- | --- | --- | --- | --- |
|  |  | |  |  | “Anthropometric measurements” |  |  |
|  |  | |  |  | OR |  |  |
|  |  | |  |  | “Body Mass index” |  |  |
|  |  | |  |  | OR |  | “Intervention” |
|  |  | |  |  | Overweight |  | OR |
|  |  | |  |  | OR |  | "Randomized clinical trial" |
| Child* |  | |  |  | Obesity |  | OR |
| OR |  | |  |  | OR |  | "Randomised clinical trial" |
| Adolescen* |  | |  |  | “Excess weight” |  | OR |
| OR | AND | | “Mediterranean diet” | AND | OR | AND | "Randomized controlled trial" |
| Youth* |  | |  |  | Adiposity |  | OR |
| OR |  | |  |  | OR |  | "Randomised controlled trial" |
| Teen* |  | |  |  | “Abdominal obesity” |  | OR |
|  |  | |  |  | OR |  | “RCT” |
|  |  | |  |  | “Body fat” |  | OR |
|  |  | |  |  | OR |  | “Clinical trial” |
|  |  | |  |  | “Fat mass” |  |  |
|  |  | |  |  | OR |  |  |
|  |  | |  |  | “High trunk fat mass” |  |  |

**Table S1**. Search strategy for MEDLINE.
