# Supplementary material for: Mediterranean Diet-Based Interventions to Improve Anthropometric and Obesity Indicators in Children and Adolescents: A Systematic Review with Meta-Analysis of Randomized Controlled Trials
Source: Adv Nutr. 2023 Apr 29;14(4):858–69. doi: 10.1016/j.advnut.2023.04.011 (PMC10334150; doi:10.1016/j.advnut.2023.04.011)
Supplement: Multimedia component 12 [file mmc12.docx]

**Table S2.** Excluded studies and reasons for exclusion.

| Atristain, E. L., Garcia, L. H., Bastarrika, I., Peña, E. M., Losa, J. A., & Alfonso, L. G. (2017). Aplicación del programa “Niños en movimiento” a nivel de una comarca: Primeros datos. 3, 8. |
| --- |
| Reason for exclusion: Quasi-experimental study. |
| Bacopoulou, F., Landis, G., Rentoumis, A., Tsitsika, A., & Efthymiou, V. (2017). Mediterranean diet decreases adolescent waist circumference. European Journal of Clinical Investigation, 47(6), 447-455. https://doi.org/10.1111/eci.12760 |
| Reason for exclusion: Quasi-experimental study. |
| Bibiloni, M. del M., Fernández-Blanco, J., Pujol-Plana, N., Surià Sonet, S., Pujol-Puyané, M. C.,  Mercadé Fuentes, S., Ojer Fernández de Soto, L., &amp; Tur, J. A. (2019). Reversión de sobrepeso y  obesidad en población infantil de Vilafranca del Penedès: Programa ACTIVA’T (2012). Gaceta  Sanitaria, 33(2), 197–202. https://doi.org/10.1016/j.gaceta.2017.10.002 |
| Reason for exclusion: Both groups with MD-based intervention. |
| Boqué, N., Tarro, L., Rosi, A., Torrell, H., Saldaña, G., Luengo, E., Rachman, Z., Pires, A., Tavares, N. T., Pires, A. S., Botelho, M. F., Mena, P., Scazzina, F., Del Rio, D., & Caimari, A. (2021). Study Protocol of a Multicenter Randomized Controlled Trial to Tackle Obesity through a Mediterranean Diet vs. A Traditional Low-Fat Diet in Adolescents: The MED4Youth Study. International Journal of Environmental Research and Public Health, 18(9). https://doi.org/10.3390/ijerph18094841 |
| Reason for exclusion: Study protocol. |
| Bruñó, A., Escobar, P., Cebolla, A., Álvarez-Pitti, J., Guixeres, J., Lurbe, E., Baños, R., & Lisón, J. F. (2018). Home-exercise Childhood Obesity Intervention: A Randomized Clinical Trial Comparing Print Versus Web-based (Move It) Platforms. Journal of Pediatric Nursing, 42, e79-e84. https://doi.org/10.1016/j.pedn.2018.04.008 |
| Reason for exclusion: Quasi-experimental study. |
| Calatayud-Sáez, F. M., & Calatayud Moscoso del Prado, B. (2020). Eficacia de la recomendación de un patrón de dieta mediterránea en preescolares con sobrepeso y obesidad. Acta pediatr. esp, 78(3/4), e101-e110. |
| Reason for exclusion: Quasi-experimental study. |
| Cobos-Palacios, L., Muñoz-Úbeda, M., Gallardo-Escribano, C., Ruiz-Moreno, M. I., Vilches-Pérez, A., Vargas-Candela, A., Leiva-Gea, I., Tinahones, F. J., Gómez-Huelgas, R., & Bernal-López, M. R. (2022). Adipokines Profile and Inflammation Biomarkers in Prepubertal Population with Obesity and Healthy Metabolic State. Children (Basel, Switzerland), 9(1), 42. https://doi.org/10.3390/children9010042 |
| Reason for exclusion: Quasi-experimental study. |
| Devecchi, A., Bo, S., De Carli, L., Breda, E., Ponzo, V., & Pezzana, A. (2022). Improve adherence to the Mediterranean diet through an innovative app: A pilot study. Nutrition & Food Science, ahead-of-print(ahead-of-print). https://doi.org/10.1108/NFS-12-2021-0394 |
| Reason for exclusion: Anthropometric data not reported. |
| Efthymiou, V., Charmandari, E., Vlachakis, D., Tsitsika, A., Pałasz, A., Chrousos, G., & Bacopoulou, F. (2021). Adolescent Self-Efficacy for Diet and Exercise Following a School-Based Multicomponent Lifestyle Intervention. Nutrients, 14(1), 97. https://doi.org/10.3390/nu14010097 |
| Reason for exclusion: Quasi-experimental study. |
| Macknin, M., Stegmeier, N., Thomas, A., Worley, S., Li, L., Hazen, S. L., & Tang, W. H. W. (2021). Three Healthy Eating Patterns and Cardiovascular Disease Risk Markers in 9 to 18 Year Olds With Body Mass Index >95%: A Randomized Trial. Clinical Pediatrics, 60(11-12), 474-484. https://doi.org/10.1177/00099228211044841 |
| Reason for exclusion: Quasi-experimental study. |
| Ranucci, C., Pippi, R., Buratta, L., Aiello, C., Gianfredi, V., Piana, N., Reginato, E., Tirimagni, A., Chiodini, E., Sbroma Tomaro, E., Gili, A., De Feo, P., Fanelli, C., & Mazzeschi, C. (2017). Effects of an Intensive Lifestyle Intervention to Treat Overweight/Obese Children and Adolescents. BioMed Research International, 2017, 8573725. https://doi.org/10.1155/2017/8573725 |
| Reason for exclusion: Anthropometric data not reported. |
| Roccaldo, R., Censi, L., D’Addezio, L., Berni Canani, S., & Gennaro, L. (2017). A teachers’ training program accompanying the «School Fruit Scheme» fruit distribution improves children’s adherence to the Mediterranean diet: An Italian trial. International Journal of Food Sciences and Nutrition, 68(7), 887-900. https://doi.org/10.1080/09637486.2017.1303826 |
| Reason for exclusion: Anthropometric data not reported. |
| Roset-Salla, M., Ramon-Cabot, J., Salabarnada-Torras, J., Pera, G., & Dalmau, A. (2016). Educational intervention to improve adherence to the Mediterranean diet among parents and their children aged 1-2 years. EniM clinical trial. Public Health Nutrition, 19(6), 1131-1144. https://doi.org/10.1017/S1368980015002219 |
| Reason for exclusion: Anthropometric data not reported. |
| Tapia-Serrano, M. A., Sevil-Serrano, J., Sánchez-Oliva, D., Vaquero-Solís, M., & Sánchez-Miguel, P. A. (2022). Effects of a school-based intervention on physical activity, sleep duration, screen time, and diet in children. Revista de Psicodidáctica (English Ed.), 27(1), 56-65. https://doi.org/10.1016/j.psicoe.2021.06.001 |
| Reason for exclusion: Anthropometric data not reported. |
| Tárraga López, P. J., Tárraga Marcos, M. L., Panisello Royo, J. M., Rosich Domenech, N., Castell Panisello, E., Carbayo Herencia, J. A., Tárraga López, P. J., Tárraga Marcos, M. L., Panisello Royo, J. M., Rosich Domenech, N., Castell Panisello, E., & Carbayo Herencia, J. A. (2017). Resultados de una intervención motivacional con niños obesos o con sobrepeso y sus familias: Estudio piloto. Revista Española de Nutrición Humana y Dietética, 21(4), 313-319. https://doi.org/10.14306/renhyd.21.4.337 |
| Reason for exclusion: Quasi-experimental study. |
