# Supplementary material for: Mediterranean Diet-Based Interventions to Improve Anthropometric and Obesity Indicators in Children and Adolescents: A Systematic Review with Meta-Analysis of Randomized Controlled Trials
Source: Adv Nutr. 2023 Apr 29;14(4):858–69. doi: 10.1016/j.advnut.2023.04.011 (PMC10334150; doi:10.1016/j.advnut.2023.04.011)
Supplement: Multimedia component 13 [file mmc13.docx]

| **Reference (year)** | **Intervention group** | | **Control group** | |
| --- | --- | --- | --- | --- |
|  | **Diet** | **Physical activity/Physical exercise** | **Diet** | **Physical activity/Physical exercise** |
| Lisón et al. (2012)(29) | Mediterranean diet | Physical exercise | Usual care | - |
| Velázquez-López et al. (2014)(38) | Mediterranean diet | - | Standard diet | - |
| Muros et al. (2015)(46) | Nutritional education | **-** | **-** | **-** |
| Peñalvo et al. (2015)(39) | Nutritional education | - | Usual care | - |
| Akdemir et al. (2017)(32) | Nutritional education | - | - | - |
| Bibiloni et al. (2017)(33) | Nutritional education | - | Usual care | - |
| Gómez et al. (2018)(36) | Nutritional education | Physical activity promotion | - | - |
| Ojeda-Rodríguez et al. (2018)(40) | Mediterranean diet | Physical activity promotion | Standard diet | Physical activity promotion |
| Akbulut et al. (2021)(44) | Mediterranean diet | Physical exercise | Low fat diet | Physical exercise |
| Fernández-Ruiz et al. (2021)(35) | Mediterranean diet | Physical activity promotion | Usual care | - |
| Prieto-Zamorano et al. (2021)(41) | Nutritional education | **-** | **-** | **-** |
| Yurtdaş et al. (2021)(48) | Mediterranean diet | - | Low fat diet | **-** |
| Blancas-Sánchez et al. (2022)(47) | Nutritional education | - | Usual care | - |
| Martíncrespo-Blanco et al. (2022)(37) | Nutritional education | **-** | **-** | **-** |
| Asoudeh et al. (2023)(45) | Mediterranean diet | - | Usual care | - |

**Table S3.** Summary of the activities carried out in the different studies analyzed.
