# Supplementary material for: Mediterranean Diet-Based Interventions to Improve Anthropometric and Obesity Indicators in Children and Adolescents: A Systematic Review with Meta-Analysis of Randomized Controlled Trials
Source: Adv Nutr. 2023 Apr 29;14(4):858–69. doi: 10.1016/j.advnut.2023.04.011 (PMC10334150; doi:10.1016/j.advnut.2023.04.011)
Supplement: Multimedia component 14 [file mmc14.docx]

**Table S4**. Detailed information about the randomized controlled trials.

| **Authors (year)** | **Description of the intervention** |
| --- | --- |
| Lisón et al. (2012)(29) | IG: Study volunteers belonging to both intervention and their parents jointly attended two 1-hour educational sessions conducted by 2 pediatricians at the Hospital. The dietary intervention focused on the promotion of the MD. This diet was specifically devised for children by one nutritionist. Total fat in this diet is 25% to 35% of the caloric intake, with saturated fat at 8% or fewer of the total calories. Families were provided with additional nutritional instruction, including interpretation of food labels and shopping, and were taught stimulus control to reduce access to high-calorie foods and increase access to healthy lower-calorie foods.  lower-calorie foods. Participants from hospital clinic group were provided with 5 supervised exercise sessions per week for 6 months (120 sessions). The participants and their parents were strongly advised to attend a minimum of 3 sessions per week (minimum attendance rate). Subjects were made to understand that “three” was the minimum required number of sessions per week to improve body composition. Exercise training was conducted at the hospital by a physical education instructor. Each session lasted 60 minutes, during which time 5 minutes were allocated for warming up and cooling down (stretching), 35 minutes were allocated to moderate aerobic activity, and 20 minutes to resistance training (low-load, high-repetition exercises).  CG: Control group participants were instructed about diet and other lifestyle changes during their regular visits to the hospital, but neither received the exercise nor the nutrition educational sessions as for the intervention groups. Control group participants maintained their usual levels of daily activity, with no additional exercise components. |
| Velázquez-López et al. (2014)(38) | IG: MD including: a) 60% of carbohydrates (50% complex and no more than 10% refined and processed sugars), 25% lipids, and 15% proteins. It was also recommended that they consume foods rich in a) essential fatty acids, such as safflower, corn, olive, and soy oils; b) omega 3 fatty acids (alpha-linolenic acid, eicosapentaenoic acid, and docosahexaenoic acid, found in foods such as salmon, mackerel, tuna, grouper, anchovies, flaxseed, canola, walnuts, and wheat germ.; c) omega 9 fatty acids (oleic and erucic acids), found in olive and canola oils, as well as Erysimum and mustard seeds; d) antioxidants, such as beta carotenes, lycopenes, vitamin A, vitamin C (found in papaya, strawberries, kiwi, oranges and other citrus fruits, green and red peppers, broccoli, spinach, and raw tomatoes), vitamin E, selenium, zinc, copper, and flavonoids (found in grapes, apples, cherries, broccoli, radishes, beets, seeds, flowers, green tea, black tea, soy, Ginkgo biloba, thistle, and cranberries); e) fiber (found in fruits and cereals). The MSD group received specific menus including the aforementioned food items. To determine the participants’ typical diet prior to the intervention, a 24-hour recalls was used.  CG: Standard diet. |
| Muros et al. (2015)(46) | IG: The nutritional education sessions informed participants about the benefits of a MD. Nutritional education involved both parents and students. For parents, there were 6 classes of nutritional education, each lasting approximately 2 hours. One session was provided each week for the first 6 weeks of intervention. Either 1 or both parents could attend the sessions. For children, there were 2 nutritional education sessions during school hours (tutorial hours), each lasting approximately 1 hour. One session was held each week for the first 2 weeks of intervention.  CG: This group continued with their usual activities. |
| Peñalvo et al. (2015)(39) | IG: The Program SI! entails four lifestyle-related components: 1) Diet (including MD), 2) Physical activity, 3) Knowledge of the human body and heart, and 4) Management of emotions. While the first two components are directly linked to a healthy lifestyle, the third component is necessary to understand the concept of health, and the fourth aims to prevent the development of behavioral disorders and drug abuse. The Program SI! is based on three domains of behavior development: 1) Knowledge, 2) Attitudes, and 3) Habits. The Program targets four population strata: 1) children, 2) parents, 3) teachers, and 4) the school environment.  CG: Usual care. |
| Akdemir et al. (2018)(32) | IG: Educational activities focusing on “healthy nutrition and active lifestyle” as well as the “causes of and preventive strategies for obesity” were provided to students and their families. In addition, in the intervention school, fresh fruits were sold at the school restaurant daily.  CG: No interventions were performed and routine educational activities were maintained. |
| Bibiloni et al. (2017)(33) | IG: For schoolchildren, the activities were divided into three 15-minute blocks: a) cartoon film based on respect, sustainability, intergenerational and intercultural dialog, recognition of local products and Mediterranean culture and culinary tradition; b) review of the theme (reinforcing the concepts explained through the characters in the film); c) filling out a worksheet related to the theme of the film (coloring, performing some kind of mathematical calculation, a crossword puzzle, etc.). For parents/families, three educational sessions were held during the course, with the help of a digital presentation. The sessions consisted of a presentation on child nutrition (approximately 30 minutes), followed by a question-and-answer session.  CG: Usual care. |
| Gómez et al. (2018)(36) | CG: The theoretical framework of the program was based on the attitude-social influence-self-efficacy model, social marketing strategies used in the public health field, and the community-based intervention guidelines for obesity prevention. In addition, the emotional approach was an important element considered in the development of each health promotion material or activity. To reinforce the health messages conveyed through the community activities, pedagogical activities, and educational material, a paper folding game was distributed each year. Some contents were related to MD and physical activity.  CG: Participants did not receive any type of information or intervention. |
| Ojeda-Rodríguez et al. (2018)(40) | IG: The intensive care group was advised to follow a moderately hypocaloric MD. Participants were encouraged to accumulate an extra time of 200 min of physical activity per week at 60–75% of their maximum heart rate. On the first visit, intensive care participants were prescribed to follow a diet based on a fixed full-day meal plan. The percentage of calorie restriction varied from 10% to 40% of total energy intake depending on the degree of obesity presented, which did not interfere with the children’s body growth. In all cases, the caloric ranges of diets were between 1300 and 2200 kcal. The diet was distributed into five main meals: breakfast providing 20%, morning snack 5–10%, lunch 30–35%, afternoon snack 10–15% and dinner 20–25% of total energy. The distribution of carbohydrate, fat and proteins was 55%, 30% and 15% of total energy, respectively.  CG: The usual group received standard pediatric recommendations on healthy diet. Participants were encouraged to accumulate an extra time of 200 min of physical activity per week at 60–75% of their maximum heart rate. |
| Fernández-Ruiz et al. (2021)(35) | IG: The I^2^AO^2^ family program focused on nutritional treatment and physical activity. The nutritional approach followed the MD model, characterized by increased consumption of olive oil, whole grains, legumes, fruits, and vegetables, moderate intake of dairy products and fish, and reduced red meat intake. Because of the age of the participants children, we did not impose any caloric restrictions and relied only on establishing healthy nutrition based on the MD. Dietary changes were based on nutritional education for both the children and parents by using games, meal preparation, considering the preferences of the children, and always in a relaxed and playful environment. The program was conducted in kitchen facilities suitable for conducting a workshop at one of the schools and focused on producing healthy and attractive recipes for the whole family (over a total of 10 sessions). Some activities were also conducted to intervene in sedentary lifestyles consisting of three weekly sessions of physical activity for 60 minutes; on 2 days the children exercised with their peers, and on the 3rd day they exercised with their guardians. The training sessions began with stretching exercises (10 minutes), followed by 30 minutes of age adjusted aerobic work and 20 minutes of play with a break at the end (120 sessions in total).  CG: The activities carried out by the research team with the families were the standard anthropometric and clinical evaluations carried out at the school. |
| Prieto-Zamorano et al. (2021)(41) | IG: Two 50-minute interventions were made for the experimental group, together with a MD informative session for the parents of this group. A theoretical-participative (PowerPoint) methodology was used in the first intervention ‘‘MD and healthy lifestyles’’, to encourage the acquisition of knowledge and a gamification activity (Kahoot) to enhanceit. During the second intervention entitled ‘‘school play-ground guidance’’, both classes of the experimental group, each in turn, were divided into pairs, and had to find different foods on a map of the dietary pyramid at different points in the playground and write them down on a control sheet.  CG: Participants did not receive any type of information or intervention. |
| Martíncrespo-Blanco et al. (2022)(37) | IG: In 45-min sessions per week, all children in the school participated in a school garden care activity, which consisted of cleaning and preparing the soil and then planting seeds of different seasonal vegetables, observing their growth, and taking care of the maintenance, watering, and cleaning of the plot. During the work in the garden, we talked to the children about how food is produced, what types of food we eat and the healthy food pyramid. They explained where fruit and vegetables are found, their importance in our daily diet and how much we should eat, focusing also on the origin of milk, fish, meat, and pulses and how often we should eat each of them. They worked on the importance of having a varied diet, how many meals a day and what they are called. During the work in the garden, they also talked about the composition of breakfast, hygiene when preparing food and hygiene when eating it, as well as fast food. After each of the sessions held with the children at school, they were given an information sheet for their parents on the topic they had dealt with that day. Along with this information, they were also given a worksheet with different drawing activities to do at home with the rest of their family. The information sheets and worksheets used the food pyramid as a basis: the first one contained information for parents on the pyramid and a drawing to color together with the children; in the next one the information focused on the necessary water consumption accompanied by drawings to cut out with the children regarding hydration according to the weather conditions and thus start decorating the pyramid. In the following sessions information was given on each step of the pyramid accompanied by drawings to color, cut out and paste with the children at each level until the pyramid was completed. Finally, the pyramids were laminated to have an individual breakfast tablecloth.  CG: In sessions of 40 min per week, participants received the usual instruction corresponding to the human body and health module. |
| Akbulut et al. (2021)(44) | IG: The Mediterranean Diet had a target macronutrient composition of 35–40% fat (with <10% of energy constituting saturated fat), 40–44% carbohydrate, and 20% protein. The children participated in a 12-week exercise program for 30–45 min/3 days in the first two weeks and for 60 min/4–5 days in the following weeks.  CG: The low-fat diet had a target macronutrient composition of 55% of energy from carbohydrates, 20–25% from fat (with <10% of energy consisting of saturated fat), and 20–25% from protein. Nutrition education focused on the selection of foods containing 3 g of fat/serving, limiting added fats, and using low-fat meal preparation strategies. Parents were instructed to provide sample quantities of grains, vegetables, fruits, lean meats, and low-fat dairy products and to restrict high fat foods. The children participated in a 12-week exercise program for 30–45 min/3 days in the first two weeks and for 60 min/4–5 days in the following weeks. |
| Yurtdaş et al. (2021)(48) | IG: Target macronutrient energy contributions were 40% from carbohydrates, 35%–40% from fat (with <10% from saturated fat), and 20% from protein. Participants in this group were instructed to consume fish and, legumes at least 2–3 times a week, and walnuts (20 g/day), and olive oil (30–45 g/day) every day, in accordance with the MD model. MD was designed to be non-calorie restricted.  CG: Target macronutrient energy contributions for the low-fat diet were 50%–60% from carbohydrates, <30% from fat (with <10% from saturated fat), and 20% from protein. Participants in this group were especially recommended to consume low-fat foods.14,15 Dietary principles to be followed in non-alcoholic fatty liver disease such as restricting saturated fat intake, avoiding processed/packaged products, alcohol, instant beverages, carbonated/sugary drinks, and sweets containing high amounts of sugar and fat, were explained to adolescents in both diet groups by the same research dietitian. Low-fat diet was designed to be non-calorie restricted. |
| Blancas-Sánchez et al. (2022)(47) | IG: Received targeted nutritional education, with specific recommendations on the MD. During the different visits, they received an evaluation of dietary adherence, the resolution of doubts about diet and food, and nutritional reinforcement for the children and families.  CG: Only received an information sheet with standardized recommendations for healthy eating, called the Decalogue of Healthy Eating. |
| Asoudeh et al. (2023)(45) | IG: The intervention in the study was MD. Accordingly, authors first computed energy requirements for each study participant based on his/her weight by adding 2 kcal per gram of weight gain. Then, macronutrient composition was determined, in which they planned each menu to have 53–58% of energy from carbohydrates, 15–18% from proteins, and 26–30% from dietary fats. In each dietary menu, increased consumption of olive oil, fruit, vegetables, and low-fat dairy products was recommended while reducing total saturated fat intake and cholesterol intake was requested. Participants were also requested to replace refined grains with dietary sources of whole grains. In addition, low consumption of sweets and red meat was recommended in these dietary menus. Along with these menus, each participant was given an Exchange list in which the exchanges for food items included in the menus were given. Participants were instructed how to use these exchange lists.  CG: Participants were only advised verbally to follow healthy eating tips recommended according to the usual Iranian diet. |

CG, control group; IG, intervention group. MD, Mediterranean diet.
