# Supplementary material for: Mediterranean Diet-Based Interventions to Improve Anthropometric and Obesity Indicators in Children and Adolescents: A Systematic Review with Meta-Analysis of Randomized Controlled Trials
Source: Adv Nutr. 2023 Apr 29;14(4):858–69. doi: 10.1016/j.advnut.2023.04.011 (PMC10334150; doi:10.1016/j.advnut.2023.04.011)
Supplement: Multimedia component 15 [file mmc15.docx]

**Table S5.** Subgroup analyses according to type intervention.

| **Variables** |  | **Only nutritional education** | | | | | **Only MD** | | | | | **MD or nutritional education and physical activity or exercise** | | | | |  |
| --- | --- | --- | --- | --- | --- | --- | --- | --- | --- | --- | --- | --- | --- | --- | --- | --- | --- |
|  | **#** | | ***d*** | **LLCI** | **ULCI** | ***I*^2^** | **#** | ***d*** | **LLCI** | **ULCI** | ***I*^2^** | **#** | ***d*** | **LLCI** | **ULCI** | ***I*^2^** | ***p*** |
| **BMI** | **7** | | −0.12 | −0.32 | 0.08 | 85.05 | **3** | −0.19 | −0.61 | 0.24 | 8.20 | **5** | −0.17 | −0.51 | 0.16 | 61.60 | 0.90 |
| **WC** | **4** | | 0.05 | −0.04 | 0.14 | 19.61 | **3** | −0.22 | −0.48 | 0.05 | 1.45 | **4** | −0.33 | −1.05 | 0.39 | 86.27 | 0.09 |
| **WHtR** | **3** | | 0.05 | −0.25 | 0.35 | 78.92 | **3** | −0.24 | −0.49 | 0.01 | 1.05 | **4** | −0.29 | −1.27 | 0.68 | 92.70 | 0.25 |
| **% OB** | **7** | | 0.06 | −0.07 | 0.19 | 66.57 | **1** | 0.00 | 0.00 | 0.00 | NA | **4** | 0.28 | −0.01 | 0.57 | 89.44 | 0.11 |
| **% AO** | **3** | | 0.01 | −0.08 | 0.09 | 35.96 | **1** | 0.00 | 0.00 | 0.00 | NA | **3** | 0.05 | −0.22 | 0.31 | 79.57 | 0.88 |

AO, abdominal obesity; BMI, body mass index; ES, effect size; LLCI, lower limit confident interval; NA, not available; OB, obesity; ULCI, upper limit confident interval; WC, waist circumference; WHtR, waist-to-height ratio. #, number of studies examined.
